# Additive Effect of Spinal Cord Volume, Diffuse and Focal Cord Pathology on Disability in Multiple Sclerosis

**DOI:** 10.3389/fneur.2019.00820

**Published:** 2019-08-06

**Authors:** Michaela Andelova, Tomas Uher, Jan Krasensky, Lukas Sobisek, Eliska Kusova, Barbora Srpova, Karolina Vodehnalova, Lucie Friedova, Jiri Motyl, Jana Lizrova Preiningerova, Eva Kubala Havrdova, Dana Horakova, Manuela Vaneckova

**Affiliations:** ^1^Department of Neurology, First Faculty of Medicine, Center of Clinical Neuroscience, Charles University and General University Hospital in Prague, Prague, Czechia; ^2^Department of Radiology, Charles University in Prague, 1st Faculty of Medicine and General University Hospital, Prague, Czechia; ^3^Endowment Fund IMPULS, Prague, Czechia

**Keywords:** magnetic resonance imaging, spinal cord, focal lesions, diffuse abnormalities, spinal cord reserve

## Abstract

**Introduction:** Spinal cord (SC) pathology is strongly associated with disability in multiple sclerosis (MS). We aimed to evaluate the association between focal and diffuse SC abnormalities and spinal cord volume and to assess their contribution to physical disability in MS patients.

**Methods:** This large sample-size cross-sectional study investigated 1,249 patients with heterogeneous MS phenotypes. Upper cervical-cord cross-sectional area (MUCCA) was calculated on an axial 3D-T2w-FatSat sequence acquired at 3T using a novel semiautomatic edge-finding tool. SC images were scored for the presence of sharply demarcated hyperintense areas (focal lesions) and homogenously increased signal intensity (diffuse changes). Patients were dichotomized according EDSS in groups with mild (EDSS up to 3.0) and moderate (EDSS ≥ 3.5) physical disability. Analysis of covariance was used to identify factors associated with dichotomized MUCCA. In binary logistic regression, the SC imaging parameters were entered in blocks to assess their individual contribution to risk of moderate disability. In order to assess the risk of combined SC damage in terms of atrophy *and* lesional pathology on disability, secondary analysis was carried out where patients were divided into four categories (SC phenotypes) according to median dichotomized MUCCA and presence/absence of focal and/or diffuse changes.

**Results:** MUCCA was strongly associated with total intracranial volume, followed by presence of diffuse SC pathology, and disease duration. Compared to the reference group (normally appearing SC, MUCCA>median), patients with the most severe SC changes (SC affected with focal and/or diffuse lesions, MUCCA<median) had an almost 5-times higher risk of having moderate disability (OR 4.75, 95% CI 3.07–7.49, *p* < 0.001). Patients with normally appearing SC and MUCCA below the median had a 2-fold increased risk of being in the moderate disability group when compared to the reference patients (OR 2.15, 95% CI 1.26–3.67, *p* < 0.001). In contrast, patients with MUCCA above the median with SC lesions/diffuse changes did not differ significantly from the reference group.

**Conclusion:** Low cervical SC volume is a strong independent predictor of physical disability in MS patients. The contribution of focal SC lesions and diffuse changes to the worse disability outcomes is limited and present especially in patients with low SC volume.

## Introduction

Multiple sclerosis (MS) is a chronic immune-mediated inflammatory and neurodegenerative disease that affects the central nervous system. The spinal cord (SC) is heavily affected in patients with MS and its involvement contributes substantially to disease progression (1, 2). Particularly at more advanced stages of MS, the dominant clinical manifestation of SC pathology resembles chronic progressive myelopathy (3). Previously, SC volume as measured on MRI was associated with disability independent from brain volume and intracerebral lesion load (4–6).

SC abnormalities are reported in between 39 and 97% of MS patients, depending on the MRI technique, and MS phenotype (7–9). Most SC lesions present as focal, well-demarcated T2-hyperintense wedge-shaped or nodular areas. In contrast to focal lesions, diffuse SC abnormalities, which are defined as poorly delineated hyperintense areas as seen on proton-density, and T2-weighted images, represent a different type of SC involvement. Diffuse SC abnormalities in patients with MS have been associated with SC atrophy, and higher disability, predominantly in patients with progressive MS (4, 10, 11). Reported correlations between conventional T2 lesion number or lesion load in SC and disability are however poor (12, 13), and focal lesions are not associate with local atrophy (14).

Besides focal and diffuse changes, SC volume has been a focus of MRI studies for more than 20 years starting with Losseff et al. (15) showing an association between SC volume reduction on MRI, and disability. Since then, numerous studies have applied edge finding techniques, voxelwise mapping, or active surface models to measure SC cross-sectional area as a surrogate of SC volume. Despite the clear association between SC volume loss and physical disability, the relationship between SC volume, focal demyelinating lesions, and diffuse SC abnormalities is not well-understood, and SC volume measurements are not used in either disease monitoring in clinical practice or as an outcome in clinical trials. A potential issue encountered by studies based solely on SC volume measurement, is the presence of pathological features which can increase tissue volume, e.g., inflammation with edema and gliosis. This could potentially hide the effects of demyelination and axonal loss (16).

We hypothesize that although SC volume might be the most relevant contributor to physical disability in relapsing-remitting MS, combining SC measurements with lesional SC imaging outcomes, such as focal SC lesions, and diffuse SC changes, may further improve predictability of disability levels. To test our hypothesis, we estimated the mean upper cervical cord cross sectional area (MUCCA) as a proxy for SC volume, and assessed the presence of focal and diffuse SC abnormalities in a large group of MS patients using 3T SC imaging acquired in a real-world settings as part of routine clinical monitoring. In addition, we investigated the relationship between SC pathology measures and physical disability.

## Materials and Methods

### Patients

In this study, we included 1,249 consecutive MS patients with clinically isolated syndrome (*n* = 169), relapse-remitting MS (*n* = 954) and early secondary progressive MS (*n* = 126) from the Center for Demyelinating Disorders, Department of Neurology in the First Faculty of Medicine and General University Hospital in Prague, Czech Republic. SC MRI examination was performed as part of routine annual clinical monitoring that we perform without specific a priory research purpose. All participants of this study agreed with collecting and analyzing their clinical, immunological, and MRI data within an international, online registry and platform for collecting prospective data on patients with MS (MSBase), and within the Czech national registry of MS patients (ReMuS). Therefore, neither ethics committee approval nor separate informed consent for this study were obtained. The MRI scanning was carried out between January 2016 and May 2016. In addition to MRI, all patients underwent standardized neurological examination to obtain Expanded Disability Status Scale (EDSS; http://www.neurostatus.net) as a part of a routine clinical monitoring.

### MR Imaging Acquisition

All patients were scanned at 3T (MAGNETOM Skyra, Siemens Healthcare, Erlangen, Germany) using the same protocol. This included a 3D-T2WI-Fat-Sat sequence in transversal plane for SC volume measurement with following parameters: time to echo (TE) 133 ms, time to repetition (TR) 1,500 ms, matrix size 320 × 320 mm, flip angle (FA) 150°, slice thickness 1.0/0 mm, field of view (FOV) 200 mm) and a sagittal T2WI-Fat-Sat sequence for assessment of focal and diffuse SC lesions (TE 84 ms, TR 2,800 ms, matrix size 384 × 384 ms, FA 160°, slice thickness 2.0/0.2 mm, FOV 220 mm).

### Semi-automatic Edge Finding Tool

**Figure 1:**
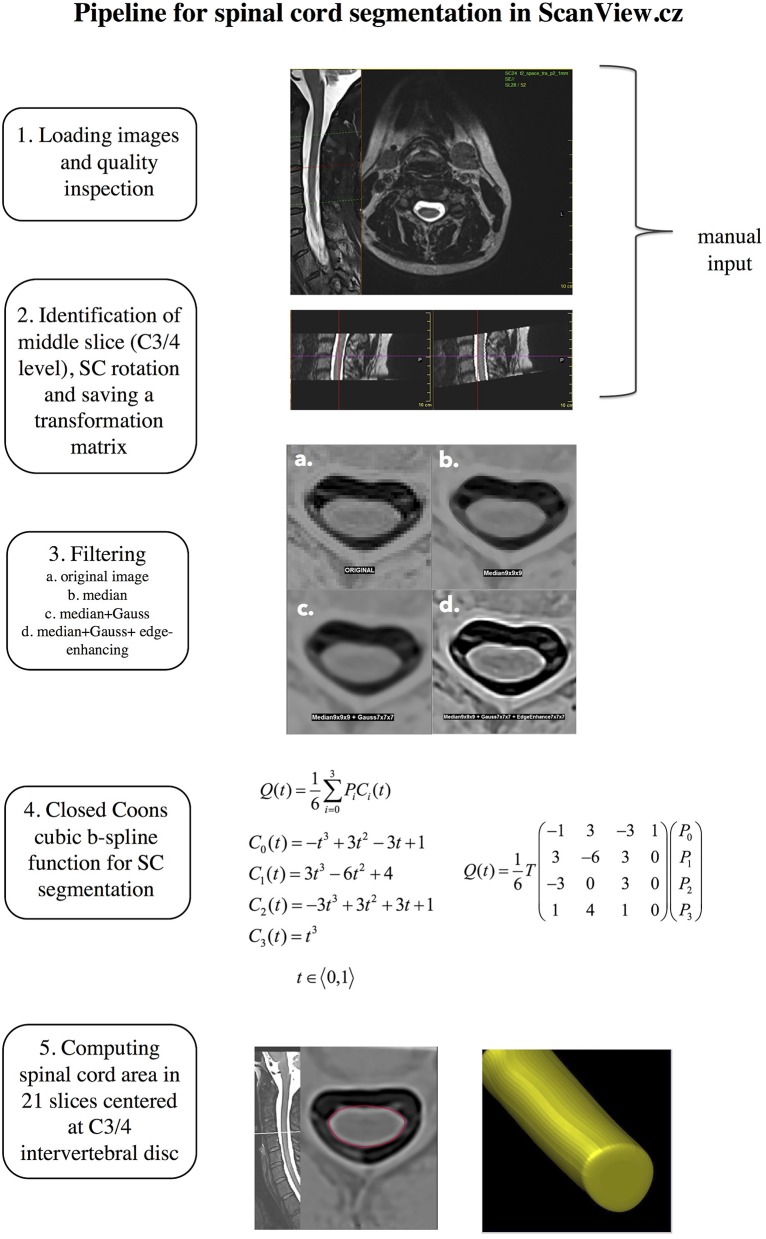
Schematic representation of the proposed pipeline for spinal cord segmentation.

The SC volume was measured semi-automatically using an in-house developed edge finding tool that is implemented in ScanView.cz (17). Briefly, in an initial manual presegmentation step, a marker is manually placed at the center of intervertebral disk C3/C4 and fixed as the “middle slice.” Subsequently, SC “straightening” takes place. The image is rotated manually to achieve a perpendicular orientation of the cord long axis relative to the axial plane (to dorsal surfaces of C3 vertebral body). This manual input requires ~2–3 min per scan. After this, a transformation matrix is saved for fully automatized subsequent steps. These steps include 1. Sub-pixel division–each voxel with original size 0.625 × 0.625 × 1 mm is divided to 27 subpixels; 2. Reversal of contrast of T2-weighted images, and applying a set of filters to reduce noise and achieve optimal contrast between SC and cerebrospinal fluid (a. median filter; b. Gaussian filter; c. edge-enhancing filter) and 4. a B-spline segmentation technique based on closed Coons cubic curve with 4 fixed control points. The closed curve representing the spinal cord consists of 6 curve segments, so that a mesh of 4 fixed and 6 independent control points build a 6 × 4 control point matrix. The B-spline basis matrix that blends the control points is shown in Figure 1. The curve segments are then put together where neighboring curve segments always share 3 control points (each segment is defined by 3 control points of the previous segment and 1 point of the following segment) in order to describe a curve that represents the spinal cord edge. Coons cubic is applied in the “middle slice” which was set manually at the C3/4 level and 10 slices in the cranial and 10 slices in the caudal direction. Finally, the area of these 21 slices is measured and the MUCCA is calculated as the sum of all areas divided by the number of slices. The steps of the proposed pipeline are shown in Figure 1.

### Spinal Cord Phenotype

**Figure 2:**
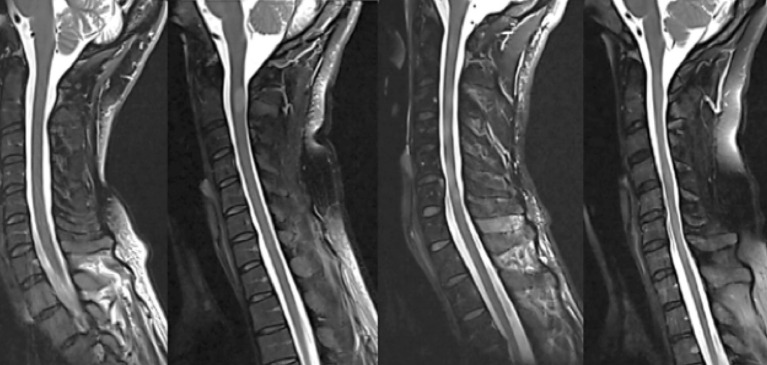
Examples of four different spinal cord patterns (from left to right): (1) normally appearing spinal cord, (2) sharply demarcated nodular T2-hyperintense lesion, (3) combination of relatively sharply demarcated lesions and diffuse T2-hyperintense abnormality, and (4) diffuse abnormality without demarcated lesions, often with noticeably irregular spinal cord diameter and atrophy.

The initial description of SC abnormalities was performed by different neuroradiologists as part of routine clinical reporting. Subsequently, a neurologist with experience in neuroradiology (M.A.) reviewed the radiological report and in case of disagreement, the images were reviewed by another experienced neuroradiologist (M.V.) to make the final conclusion. Sagittal T2WI Fat-Sat was used to assess the number and location of vertebral segments involved in focal lesions, and/or diffuse changes. The classification of SC abnormalities was based on the work by Lycklama á Nijeholt et al. (9) defining 3 groups of SC changes based on T2/proton density appearance: (1) Focal lesions defined as sharply demarcated T2-hyperintense areas, (2) If the T2-weighted images showed homogenously increased signal throughout the majority of cervical and thoracic levels, the change was assessed as “diffuse abnormality,” and (3) Sharply demarcated hyperintense lesions in the background of homogenously increased signal were recorded as “focal lesions and diffuse abnormalities.” Besides these three groups, a fourth category was classified: 4. normally appearing SC. Figure 2 shows examples of the SC pathology patterns. Subsequently, we combined the information about the presence/absence of lesions, and diffuse changes with MUCCA. Due to frequent overlap between multiple focal and diffuse SC changes, we decided to include both parameters into one category. As our work was of exploratory nature and no healthy control data were available, we used sex-specific medians of MUCCA as the cut-off value. The combination of volumetric (MUCCA) dichotomized information and presence or absence of focal, and/or diffuse SC lesions resulted in four SC “phenotypes” that were used for ordinal regression analysis (see below): (1) Normally appearing SC + MUCCA above the median; (2) SC affected with focal and/or diffuse lesions + MUCCA above the median; (3) normally appearing SC + MUCCA below the median; (4) SC affected with focal and/or diffuse lesions + MUCCA below the median.

### Statistical Analysis

SPSS software (Version 20, Chicago, IL, USA) was used for the statistical analyses.

#### Reliability

First, the reliability of the employed processing method was assessed. Two raters (E.K. and M.A.), performed the “presegmentation” steps discussed above) in 20 randomly selected patients. Each presegmentation was done twice per rater, with a 1 week interval. Intra- and interrater reliability was assessed by model 2 intraclass correlation coefficients (ICC). ICC estimates and their 95% confidence intervals were calculated based on a mean-rating, absolute-agreement, 2-way mixed-effects model.

#### Determinants of Spinal Cord Volume

To determine the potential effect of sex, age, disease duration, total intracranial volume (TIV), focal SC lesions, and diffuse SC changes on MUCCA, we used analysis of covariance (ANCOVA).

#### Relationship Between SC Phenotype, SC Volume, and Physical Disability

The distribution of particular SC changes between patients with mild and moderate disability was investigated by means of chi-square test. Spearman rank correlation test was used to assess associations between SC lesion number and clinical measures including EDSS score and FS scores, disease duration, and age. Binary logistic regression analyses were performed to assess the impact of demographic, clinical, and SC pathology measures on the physical disability defined as a dichotomous dependent variable (similarly as in Naismith et al. (18) patients were dichotomized into groups with mild disability with EDSS ≤3.0 or moderate disability with EDSS≥3.5; as only 33 patients had EDSS ≥ 6 (max. EDSS 7.0), a category with “severe disability” was not considered). Dichotomized disability was used as the dependent variable in two analyses. The independent variables of the first model were sex, age, disease duration, and SC phenotypes (4 above mentioned categories based on a combination of dichotomized MUCCA and presence of focal and/or diffuse changes). In a second model, we assessed the relative contributions of sex, age, disease duration, MUCCA (expressed as dichotomous variable below/above median), presence, and number of focal lesions and/or diffuse changes and interaction between MUCCA, focal and diffuse changes to disability. The correction for false discovery rate with *p* < 0.01 was used to minimize the false discovery rate. As our cohort consisted mainly of actively treated patients, only 55 patients fulfilled the objective criteria of secondary progression as defined by Lorscheider et al. (19), we have not analyzed the contribution of MUCCA and particular SC phenotypes in CIS, RRMS, and SPMS patients separately. Instead, we performed an identical binary logistic regression analyses as mentioned above in patients who were fully ambulatory [EDSS up to 4.0, higher and lower disability groups defined as EDSS ≤ 1.5 and EDSS >1.5) and in patients with ambulation impairment (EDSS > 4.0, higher and lower disability defined as EDSS <6.0 and EDSS ≥ 6)].

## Results

Table 1 summarizes demographic and clinical data of our cohort (for a detailed description regarding spinal cord phenotype see Methods).

**Table 1:** Demographic, clinical and MRI parameters of the cohort.

	**All patients**	**Mild disability EDSS 0–3.0**	**Moderate disability EDSS ≥ 3.5**
n patients (% women)	1,249 (71)	902 (70)	347 (75.5)
Mean age years ± SD	40.6 ± 9.7	38.8 ± 9.0	45.5 ± 7.9
Mean DD years ± SD	11.1 ± 7.7	9.36 ± 6.8	15.59 ± 7.93
Median EDSS (min.–max.)	2.5 (0–7.0)	2.0 (0–3)	4.0 (3.5–7)
MUCCA (mm^2^ ± SD)	83.95 ± 9.69	85.99 ± 8.81	78.65 ± 9.86
**Spinal cord phenotype**			
Normally appearing n (%)	424 (33.9)	346 (38.4)	78 (22.5)
Focal lesions n (%)	318 (25.5)	225 (24.9)	93 (26.8)
Diffuse changes n (%)	326 (26.1)	217 (24.1)	109 (31.4)
Diffuse changes and focal lesions n (%)	181 (14.5)	114 (12.6)	67 (19.3)

*DD, disease duration; EDSS, Expanded Disability Status Scale; MUCCA, mean upper cervical cord area; SD, standard deviation*.

### Reliability of the Spinal Cord Measurement

We found high agreement both within and between raters. For the intra-rater agreement, the average ICC of rater 1 was 0.999 (95% CI 0.997–1.0, *F* = 1,714,6, *p* < 0.001) and the average ICC of the rater 2 was 0.999 (95% CI 0.996–0.999, *F* = 1,233.66, *p* < 0.001). In the inter-rater reliability analysis, the average ICC was 0.994 (95%CI.984–0.998, *F* = 18.161, *p* < 0.001).

### Spinal Cord Pathology

**Figure 3:**
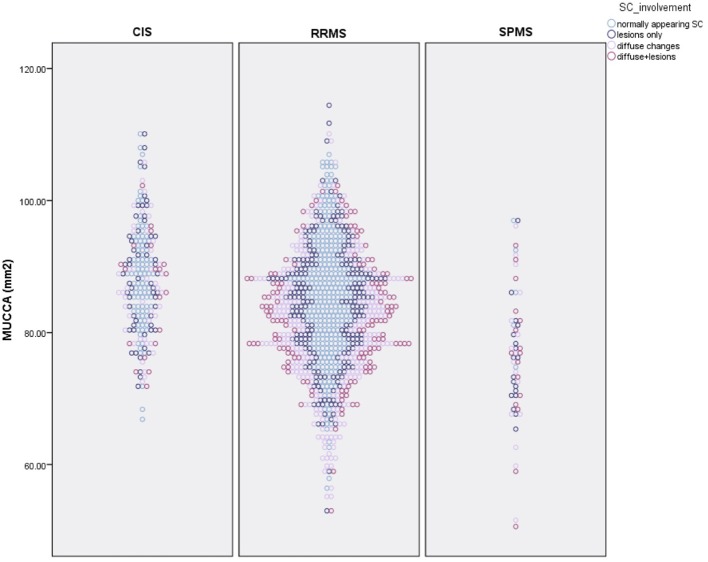
Distribution of spinal cord phenotypes in patients with CIS, RRMS, and SPMS and its association with spinal cord volume (MUCCA–mean upper cross-sectional cervical cord area).

Overall, 424 patients (33.9%) had a normally appearing SC. Of the 499 patients with focal SC pathology, there were a total of 898 focal, sharply demarcated SC lesions: 318 patients (25.5%) had focal lesions without diffuse abnormalities, and 181 patients (14.5%) had both focal lesions, and diffuse abnormalities. The majority of the lesions were located in the upper cervical cord: 26.7% of all lesions were on C1/2 level, 22.3% on C3 level, 19.5% on C4 level, 18.3% on C5 level, 13.9% on C6 level, 7.1% on C7 level, and only 7.8% on the Th1 to Th5 level. 326 patients (26.1%) had diffuse abnormalities without sharply demarcated lesions. The distribution of lesions did not differ between males and females (chi-square = 4.19, *p* = 0.24). The distribution of SC phenotypes differed significantly between patients with CIS and RRMS (chi-square = 13.68, *p* = 0.003) and RRMS and SPMS (chi-square = 17.85, *p* < 0.001). Table 2 and Figure 3 show the distribution between SC phenotypes in patients with CIS, RRMS, and SPMS, its association with MUCCA and physical disability.

**Table 2:** Distribution of SC phenotypes and its relationship to spinal cord volume (MUCCA) and disability (EDSS).

	**CIS**			**RRMS**			**SPMS**		
	**%**	**MUCCA (mm^**2**^)**	**EDSS median**	**%**	**MUCCA (mm^**2**^)**	**EDSS median**	**%**	**MUCCA (mm^**2**^)**	**EDSS median**
Normally appearing SC	44.04	88.37 ± 7.63	1.5	33.12	85.63 ± 9.04	2.0	9.09	86.32 ± 8.84	5.0
Lesions only	26.61	88.68 ± 8.96	1.5	24.82	84.40 ± 9.18	2.0	32.73	75.83 ± 7.57	5.0
Diffuse changes	20.18	87.22 ± 8.37	2.0	27.44	80.55 ± 10.27	2.5	29.09	75.80 ± 12.12	5.5
Diffuse changes+lesions	9.17	86.21 ± 8.48	2.0	14.62	82.44 ± 9.01	2.5	29.09	76.03 ± 11.17	5.5

*MUCCA, mean upper cervical cord cross-sectional area; EDSS, expanded disability status scale*.

### Determinants of Spinal Cord Volume

The raw MUCCA was lower in women than men (*p* < 0.001, mean in men: 86.96 mm^2^ [95% CI = 85.98–87.95]; in women: 82.76 mm^2^ [95% CI 82.14–83.40]). When adjusted for TIV, the sex-related difference in MUCCA disappeared (*p* = 0.243, mean in men 85.0 mm^2^ [95% CI 83.47–85.53], in women: 83.75 mm^2^ [95% CI 83.13–84.37]). TIV explained 9.4% of MUCCA variability. Disease duration and presence of diffuse SC abnormalities explained 3.8 and 3.6% of the MUCCA variability. TIV, disease duration, and diffuse SC abnormalities were independent predictors of MUCCA in a multivariable model (Table 3).

**Table 3:** Predictors of cervical spinal cord volume characterized by the mean upper cervical cord cross-sectional area.

**Variable**	**F**	**Significance**	**Partial eta squared**
Disease duration	47.711	<0.001	0.038
Age	1.121	0.290	0.001
Sex	1.889	0.170	0.002
Total intracranial volume	127.680	<0.001	0.094
Number of lesions	3.166	0.075	0.003
Diffuse SC abnormalities	45.904	<0.001	0.036
Corrected model	58.164	<0.001	0.222

*Statistical analysis was performed using multivariable ANCOVA. SC, spinal cord*.

### Relationship Between SC Pathology, SC Volume, and Physical Disability

As shown in Table 4, there was a modest association between MUCCA and EDSS (rho = −0.315), pyramidal (rho = −0.341), sensory (rho = −0.231), and bowel and bladder FS scores (rho = −0.251), cumulative relapse rate (rho = −0.279), age (rho = −0.161), and disease duration (rho = −0.272). Number of spinal cord lesions was only weakly correlated with EDSS, pyramidal FS score, and relapse rate.

**Table 4:** Spearman rank correlation coefficients indicating association between T2 hyperintense spinal cord lesion number and clinical measures.

		**EDSS**	**Pyramidal FS score**	**Sensory FS score**	**Bowel + bladder FS score**	**Annualized relapse rate**	**Cumulative relapse rate**	**Age**	**Disease duration**
Number of SC lesions	m	n.s.	n.s.	0.192^*^	n.s.	n.s.	n.s.	n.s.	n.s.
	f	n.s.	n.s.	n.s.	n.s.	n.s.	n.s.	n.s.	n.s.
	all	0.077^*^	0.059^*^	n.s.	n.s.	n.s.	0.062^*^	n.s.	n.s.
MUCCA (mm^2^)	m	−0.326^*^	−0.355^*^	−0.218^*^	−0.197^*^	n.s.	−0.280^*^	−0.155^*^	−0.277^*^
	f	−0.316^*^	−0.339^*^	−0.231^*^	−0.276^*^	n.s.	−0.267^*^	−0.156^*^	−0.265^*^
	all	−0.315^*^	−0.341^*^	−0.231^*^	−0.251^*^	n.s.	−0.279^*^	−0.161^*^	−0.272^*^

FS, functional system; MUCCA, mean upper cervical cord cross-sectional area; n.s., not significant;

* *p <0.01 after correction for false discovery rate*.

The raw MUCCA was lower in patients with EDSS ≥ 3.5 (mean = 78.86 mm^2^ [95% CI 77.72–79.64]) than in patients with EDSS ≥ 3.0 (*p* < 0.001, 85.98 mm^2^ [95% CI 85.38–86.58]). The difference in MUCCA between EDSS groups remained significant even after adjustment for TIV (*p* < 0.001, mean for EDSS ≥ 3.5 79.33 mm^2^ [95% CI 78.41–80.24], mean for EDSS up to 3.0: 85.74 mm^2^ [95% CI 85.17–86.30]).

**Figure 4:**
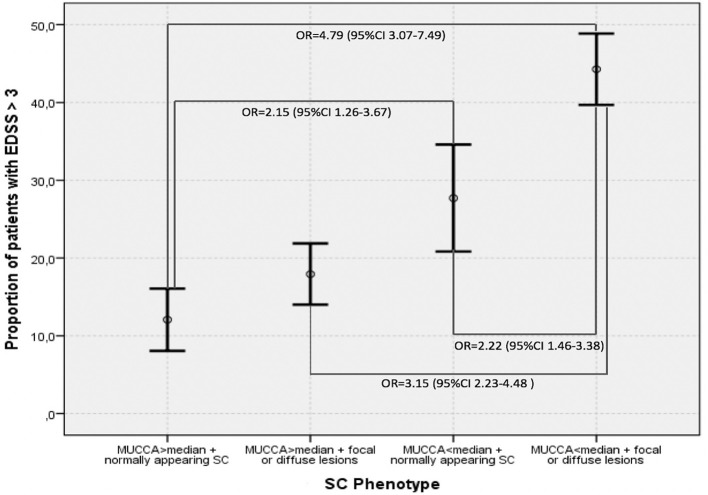
Proportions of patients with EDSS > 3.0 in different SC phenotypes defined by dichotomized magnetic resonance imaging predictors–MUCCA below and above median, presence, respectively, absence of focal lesions and diffuse changes. Means and 95% confidence intervals are shown.

Figure 4 demonstrates the proportions of patients with moderate disability (EDSS ≥3.0) in the four SC phenotype groups as defined by the dichotomized MUCCA and SC involvement by focal lesions ad/or diffuse abnormalities. Patients with the “most severe” SC changes (SC affected with focal and/or diffuse lesions + MUCCA below the median) had an almost 5-times higher risk of having moderate disability (OR 4.75, 95% CI 3.07–7.49, *p* < 0.001) compared to the reference group (normally appearing SC + MUCCA above the median). Patients with normally appearing SC below median had a 2-fold increased risk of being in the moderate disability group when compared to the reference patients (OR 2.15, 95% CI 1.26–3.67, *p* < 0.001). Patients with the “most severe” SC phenotype (SC affected with focal and/or diffuse lesions + MUCCA below the median) had a higher risk of moderate disability when compared to patients with SC lesions and/or diffuse changes with MUCCA above the median (OR 3.15, 95% CI 2.23–4.48, *p* < 0.001) and to patients with normally appearing SC and with MUCCA below the median (OR 2.22, 95% CI 1.46–3.38, *p* = 0.003). In contrast, among patients with MUCCA above the median with SC lesions/diffuse changes, the risk of having moderate disability did not differ significantly from neither the reference group, nor from the patients with normally appearing SC and MUCCA below the median. If MUCCA was above the median, patients with normally appearing SC and patients with SC affected by lesions and/or diffuse changes did not differ in terms of SC volume (MUCCA = 91.54 ± 5.92 vs. 91.33 ± 5.88), whereas in patients with MUCCA below the median, SC volume was significantly smaller in patients with focal and/or diffuse changes than in patients with normally appearing (78.0 ± 5.52 vs. 75.88 ± 6.59). Further demographic, and clinical characteristics of the four SC phenotypes are summarized in Table 5.

**Table 5:** Demographic, clinical, and MRI parameters in the four SC phenotypes.

	**MUCCA > median normally appearing SC (*n* = 257)**	**MUCCA > median with lesions/or diffuse changes (*n* = 368)**	**MUCCA < median Normally appearing SC (*n* = 166)**	**MUCCA < median with lesions/diffuse changes (*n* = 454)**
MUCCA	91.54 ± 5.92	91.33 ± 5.88	78.0 ± 5.52	75.88 ± 6.59
Age	39.47 ± 9.63	39.26 ± 9.08	43.18 ± 9.38	41.57 ± 9.89
Disease duration	8.77 ± 6.12	10.0 ± 7.64	11.29 ± 7.30	13.18 ± 8.03
EDSS	2.0 (0–6)	2.0 (0–6.5)	2.0 (0–6.5)	3.0 (0–7.0)

*MUCCA, mean upper cervical cord cross-sectional area; EDSS, Expanded Disability Status Scale. Data are presented as means (±standard deviation) except of EDSS steps that are described as median (min.–max.)*.

In a binary regression model with dichotomized disability as the dependent variable exploring the relative contribution of MUCCA, focal and diffuse SC lesions, all parameters, i.e., MUCCA (*p* < 0.001), presence of diffuse changes (*p* = 0.031) and focal lesions (*p* < 0.001) were independently associated with a higher risk of moderate disability. Seventy four percent of the disability variance was explained by disease duration and age. Among the SC parameters, dichotomized MUCCA explained about 20.1% of overall explained disability variance and, whereas the relative contributions of diffuse changes (3.4% of explained variance) and presence, respectively, the number of focal SC lesions (3.9% of explained variance) further improved the model, however, their contributions were only small (R^2^ and χ^2^ are shown in Tables 6A,B). An interaction of MUCCA, focal and diffuse pathology fell short of significance. The association of spinal cord parameters with disability in a binary regression model in patients with EDSS ≤ 4.0 and dichotomized disability (EDSS ≤ 1.5 and EDSS >1.5) was significant, but again, explained only a fraction of disability variance. 78.7% of the disability variance was explained by disease duration and age, dichotomized MUCCA explained 6.8% of disability variance, diffuse changes and lesions explained 8.9%, respectively, 5.5% of disability variance. In patients with high disability (EDSS > 4.0), SC parameters were not significantly associated with disability (EDSS up to 5.5 and EDSS ≥ 6 (data not shown).

**Table 6A:** Association between disability group and demographical, clinical and SC pathology measures.

**Predictors**	**B**	**SE**	**p**	**OR**	**95% CI for OR**	**R^**2**^**	**χ^2^ for the block**	***p*-value**
Disease duration	0.070	0.010	<0.001	1.073	1.051–1.095	0.207	192.51	<0.001
Age	0.049	0.009	<0.001	1.050	1.033–1.068			
Gender	−0.261	0.163	0.108	0.770	0.560–1.059			
**Model 1: MUCCA, dichotomized presence of diffuse changes and presence of focal SC lesions (all variables dichotomized)**								
Block1: MUCCA (below) median	1.040	0.150	<0.001	2.828	2.109–3.791	0.263	58.18	<0.001
Block 2: +Presence of diffuse changes	0.632	0.177	<0.001	1.881	1.330–2.661	0.266	4.64	0.031
Block 3: +Presence of focal lesions	0.660	0.196	0.001	1.935	1.317–2.843	0.279	11.416	0.001
**Model 2: dichotomized MUCCA, dichotomized presence of diffuse changes and absolute number of focal SC lesions**								
Block1: MUCCA (below) median	1.040	0.150	<0.001	2.828	2.109–3.791	0.263	58.18	<0.001
Block 2: + Presence of diffuse changes	0.346	0.146	0.018	1.413	1.061–1.883	0.268	4.64	0.031
Block 3: + Number of lesions	0.227	0.064	<0.001	1.255	1.107–1.423	0.279	12.38	<0.001

*MUCCA, mean upper cervical cord cross-sectional area; R2, Nagelkerke R square; χ^2^, chi square*.

**Table 6B:** Association between disability group (EDSS ≤ 1.5 and EDSS > 1.5) and demographical, clinical, and SC pathology measures in patients with EDSS ≤ 4.0.

**Predictors**	**B**	**SE**	***p***	**OR**	**95% CI for OR**	**R^**2**^**	**χ^2^ for the block**	***p*-value**
Disease duration	0.030	0.011	0.005	1.031	1.009–1.052	0.115	98.41	<0.001
Age	0.058	0.008	<0.001	1.060	1.042–1.077			
Gender	−0.172	0.142	0.227	0.842	0.637–1.113			
**Model 1: MUCCA, dichotomized presence of diffuse changes and presence of focal SC lesions (all variables dichotomized)**								
Block1: MUCCA (below) median	0.337	0.134	0.012	1.401	1.078–1.820	0.125	9.23	0.002
Block 2: +Presence of diffuse changes	0.483	0.139	<0.001	1.621	1.235–2.127	0.138	11.40	0.001
Block 3: +Presence of focal lesions	0.217	0.135	0.108	1.242	0.953–1.618	0.140	2.60	0.107
**Model 2: dichotomized MUCCA, dichotomized presence of diffuse changes and absolute number of focal SC lesions**								
Block1: MUCCA (below) median	0.335	0.134	0.012	1.399	1.076–1.818	0.125	9.226	0.002
Block 2: +Presence of diffuse changes	0.484	0.138	<0.001	1.622	1.237–2.128	0.138	11.39	0.001
Block 3: +number of focal lesions	0.179	0.064	0.006	1.196	1.054–1.357	0.146	7.961	0.005

*MUCCA, mean upper cervical cord cross-sectional area; R2, Nagelkerke R square; χ^2^, chi square*.

## Discussion

This study investigated the interaction between cervical SC volume, focal lesions, and diffuse abnormalities, and neurological disability in a large cohort of MS patients. Although both SC volume loss and SC lesional pathology were associated with physical disability, the independent contribution between these two aspects of SC damage on disability status was present only in patients with low spinal cord volume. In the whole group of patients, SC volume measured as MUCCA was a stronger predictor of physical disability than focal SC lesions or diffuse SC changes. On the contrary, in patients with lower disability (EDSS up to 4.0), presence of diffuse SC changes was associated with physical disability stronger than low SC volume.

Binary logistic regression analyses confirmed observations from previous studies that cervical SC volume was strongly associated with disability when analyzing all patients. In multivariable models with SC volume, the additive effect of focal, and/or diffuse changes was limited, and specifically, it was only evident in the group with low MUCCA. Importantly, a significant difference in MUCCA was observed between patients with diffuse, and focal changes in the group with MUCCA below the median, whereas in the group with MUCCA above median, SC phenotype was not associated with differences in SC volume. This is analogous to previous brain imaging studies showing a complementary effect between brain MS lesion accumulation and brain atrophy on physical, and cognitive disability (20, 21). Longitudinal analysis is needed to elucidate whether patients with focal and diffuse changes develop spinal cord atrophy faster than patients with a normally appearing SC. Moreover, detailed description of lesion extent and localization within SC gray matter or particular SC white matter columns would be beneficial, considering their association with poorer clinical outcome (22). A previous study has shown that the number of SC segments involved by focal lesions and the presence of diffuse SC abnormalities were the most significant predictors of cervical cord atrophy rates (23). We speculate that lesions and diffuse changes in patients with a MUCCA above the median might be less unfavorably localized and/or be less active and damaging than those in patients with MUCCA below the median. Moreover, cerebral lesion load and distribution may play a role in spinal cord atrophy as well.

It is worth mentioned that we do not understand the histopathological changes underlying SC volume loss and diffuse changes. As permanent neurological disability correlates with SC axonal loss, it seems feasible to interpret MUCCA as a marker of axonal loss. Intriguingly, in a recent post-mortem study, SC atrophy seemed to significantly underestimate the degree of axonal loss. Authors detected an association between focal demyelination and decreased axonal density, but SC cross-sectional area appeared to be a poor predictor of the axonal density (24). Similarly, diffuse SC changes are not a strong predictor of axonal loss as shown histopathologically (25) and instead reflect the extent of demyelination (26). Diffuse changes associated with the number of focal SC lesions is present already in early MS, as has been reporting using magnetization transfer ratio (MTR) (27) or they may develop from demarcated, focal lesions, are permanent, and their presence early in the relapsing-remitting MS confers unfavorable prognosis–almost 70% of patients with diffuse abnormalities reach EDSS 4.0 in 6 years as opposed to 25% with focal lesions who reach EDSS 4.0 in 11 years (28). Our results of binary logistics regression in patients with EDSS up to 4.0 might also suggest that in less disabled patients, diffuse changes are associated with higher disability even stronger than spinal cord volume, suggesting diffuse changes may be earlier marker of spinal cord damage than atrophy at least in some patients. Further studies combining imaging, and histopathology are needed to elucidate, whether characteristics of focal SC lesions and diffuse changes relate to SC atrophy. Additionally, imaging parameters reflecting different pathophysiological processes such as meningeal inflammation (29) might help to understand the association of focal SC damage, SC volume and disability. Importantly, brain regional, and global volume, and brain lesions have to be taken into account to understand the association between supra-, and infratentorial pathology.

Distinct myelitis characteristics, i.e., location, length, and enhancement pattern are crucial in differential diagnosis. Whereas, the SC lesions in MS are usually focal, involve one or two segments and are localized on the spinal cord periphery, often in the posterior and lateral parts of SC, lesions in neuromyelitis optica (NMO), and acute disseminated encephalomyelitis (ADEM) are localized centrally. Confluent longitudinal myelitis with pronounced swelling has been described in ADEM (30, 31). Recently, opticospinal demyelinating diseases associated with antibodies against myelin oligodendrocyte glycoprotein (MOG) has gained increasing attention. In anti-MOG-antibodies-associated illness, lower part of the spinal cord might be preferentially affected (lesions are predominantly in thoracic and lumbar segments as well as conus medullaris), and extensive SC atrophy has been described (32, 33). Ultimately, these different patterns of SC involvement and their association with SC volume might help us to better understand the underlying pathophysiology, i.e., distribution of specific antigens in spinal cord or type of immune response.

Whereas, previous research showed that T2-weighted imaging can detect SC lesions in as many as 90% of patients (10), we found a surprisingly large proportion of patients with a normally appearing SC. This may be due to relatively high proportion of patients with no or mild disability and a low number of patients with high disability (only 33 patients had EDSS 6.0 and higher) in our cohort. Similar results were reported in a study by Dastagir et al. (34), where normally appearing SC is described in 25% of patients with RR-RS as well as in study by Rovaris et al. (7), describing focal lesions in only 39% of patients with early MS. Moreover, as the majority of data on SC involvement in MS was obtained on 1.5T scanners, it is important to note that a higher total and thoracic SC lesion number was found in progressive MS patients when compared to relapse-remitting MS patients on 1.5T, but not 3.0T (13). In the same study, pyramidal, and bowel and bladder FS scores were significantly associated with SC lesion number at 1.5T, while correlations at 3.0 were non-significant. Authors attribute this difference to cardiac and respiratory cycle artifacts that are adversely impacting 3.0 more than 1.5T imaging. Finally, as we assessed SC lesions on a single T2WI-Fat-Sat sagittal sequence, absence of complementary PD sequence as well as absence of axial planes might result in underestimation of lesion number.

The moderate correlations between MUCCA and EDSS scores are in line with data obtained at 1.5T by Stankiewicz (13). We observed weak, but significant correlations between MUCCA, pyramidal, bowel, and bladder and sensory FS scores. Moreover, the MUCCA measurement detects the previously reported male-female differences in the raw upper cervical cord volume (4, 35, 36). After adjusting for TIV, the observed difference between males, and females was lost, supporting the importance of adjusting for TIV, and sex in MS analyses comprising SC.

We acknowledge several limitations of our study. We did not perform blinded inter-rater reliability for focal, and diffuse changes assessment. However, the scoring of changes was done stepwise and problematic cases were carefully reviewed. Another drawback of the study was the lack of a proton density sequence which is recommended for the identification of diffuse spinal cord changes. We have observed that indirect SC characteristics, such as the variability of SC diameter in absence of focal lesions, may help to distinguish diffuse changes from artifacts. Ideally, a quantitative MRI parameter such as diffusion tensor imaging or MTR (27, 37), MR spectroscopy (38) or quantitative T1 would objectivize or even quantify the severity of SC diffuse changes and increase its value as a biomarker for disability prediction and treatment response in clinical trials. Moreover, as the number and extent of SC lesions were assessed only on sagittal T2-weighted images and only in the segments C1 to Th4, the number of lesions in might be underestimated. 3D phase-sensitive inversion recovery significantly improves detection of cervical spinal cord lesions (39). A healthy control group was not included in the present study, deeming it impossible to determine sex-, and age-specific cut-offs for spinal cord volumes. Longitudinal data from age-matched healthy volunteers for establishing pathological SC atrophy cut-offs and longitudinal data from patients are currently being collected, and evaluated. As mentioned above, SC data were not analyzed in conjunction with brain lesional and volumetric parameters, deeming it impossible to compare relative contributions of brain and SC to disability.

In conclusion, the results of our study suggest that SC volume and to a lesser extent focal and diffuse SC changes, are associated with physical disability in MS patients, suggesting there is a complementary effect of lesional and atrophy SC measures in explaining disability in MS patients. This complementary effect is particularly evident in patients with small SC volume and when distinguishing between patients with mild and moderate atrophy. SC volume reduction might be therefore a MRI marker of transition toward secondary progressive disease. In patients with lower disability, diffuse changes surpass SC volume in distinguishing between patients with minimal and mild to moderate disability. This finding underlines the importance of taking different SC parameters into account in different patient populations. Studies studying a concept of a “spinal cord reserve” and identifying characteristics of focal and diffuse lesions associated with more aggressive SC volume loss are warranted.

## Data Availability

The data supporting the findings of this study are available from the corresponding author (MA), upon reasonable request.

## Author Contributions

MV, DH, TU, LS, JK, EKH, JP, and MA: design of the study. JK: semiautomatic edge-finding tool. MV, EK, and MA: manual steps in the semiautomatic spinal cord measurement and description of SC changes. BS, KV, LF, JM, and JP: clinical examination. TU, LS, and MA: statistical analysis. MA and TU: first draft and revising of the manuscript. MV, DH, TU, LS, JK, EKH, JP, BS, KV, LF, JM, and MA: article correction and final approval.

### Conflict of Interest Statement

MA received financial support for conference travel from Novartis, Genzyme, Merck Serono, Biogen Idec, and Roche. JK received financial support for research activities from Biogen Idec. TU received financial support for conference travel from Biogen Idec, Novartis, Sanofi, Roche, and Merck Serono, and speaker honoraria from Biogen Idec, Novartis, and Roche as well as support for research activities from Biogen Idec and Sanofi. LS received financial support from Novartis. BS received compensation for traveling and conference fees from Novartis, Sanofi Genzyme, Biogen Idec, Roche, and Merck as well as support for research activities from Biogen Idec. KV received compensation for traveling and conference fees from Merck, Sanofi Genzyme, Biogen Idec, and Novartis. LF received compensation for traveling, conference fees, and speaker honoraria from Biogen and Roche. JM received compensation for traveling, conference fees, and speaker honoraria from Sanofi Genzyme, Biogen, Novartis, and Merck. JP has received speaker and consulting fees from Biogen, Novartis, and Teva. EKH received speaker honoraria and consultant fees from Biogen Idec, Merck Serono, Novartis, Genzyme, and Teva, as well as support for research activities from Biogen Idec and Merck Serono. DH received compensation for travel, speaker honoraria, and consultant fees from Biogen Idec, Novartis, Merck, Bayer, Sanofi Genzyme, Roche, and Teva, as well as support for research activities from Biogen Idec. MV received speaker honoraria and consultant fees from Biogen Idec, Novartis, Sanofi Genzyme, Merck Serono, and Teva, as well as support for research activities from Biogen Idec. The remaining author declares that the research was conducted in the absence of any commercial or financial relationships that could be construed as a potential conflict of interest.
